# Development of an AI-driven chatbot for medication-assisted treatment standards in Scotland

**DOI:** 10.3389/fdgth.2026.1698657

**Published:** 2026-07-29

**Authors:** Sandra C. Nwobi, Zainab Loukil, Abbas Jawahar

**Affiliations:** 1 School of Business, Computing and Social Sciences, University of Gloucestershire, Cheltenham, United Kingdom; 2 Cyber and Technical Computing, School of Business, Computing and Social Sciences, University of Gloucestershire, Cheltenham, United Kingdom

**Keywords:** Artificial intelligence in healthcare, healthcare chatbot, Knowledge Graphs (KGs), Large Language Models (LLMs), Medication-Assisted Treatment (MAT), opioid use disorder, Retrieval-Augmented Generation (RAG), Scotland

## Abstract

**Background:**

Scotland faces a severe public health crisis with drug-related deaths reaching 267 per million people, ranking second globally after the United States. Medication-Assisted Treatment (MAT) represents a proven intervention for heroin addiction. However, healthcare professionals struggle with accessing and interpreting current MAT standards through fragmented information systems and time-consuming manual searches across multiple websites. Despite advances in healthcare chatbots leveraging Large Language Models (LLMs), no specialized systems exist to support MAT delivery or integrate advanced technologies like Retrieval-Augmented Generation (RAG) and Knowledge Graphs for addiction treatment.

**Objective:**

To develop and evaluate an AI-driven chatbot prototype that integrates LLMs, RAG, and Knowledge Graphs to enhance healthcare professionals’ access to MAT standards in Scotland, addressing current barriers in information delivery and clinical decision-making.

**Methods:**

We employed a mixed-methods approach combining a survey of 39 MAT healthcare professionals (31% response rate) and systematic literature review following PRISMA guidelines. The chatbot prototype was developed using Llama2 language model, Neo4j knowledge graphs, and custom RAG implementation. Data was ethically collected from Public Health Scotland and Healthcare Improvement Scotland websites. Performance was evaluated using BLEU and ROUGE metrics, with prototype deployment via Streamlit interface.

**Results:**

Survey findings revealed significant challenges with current communication methods: only 5 of 39 respondents rated existing systems as “exceptional,” while 17 rated them as “average” or below. Primary challenges included decentralized information (n=13) and time-consuming access processes (n=8). Literature review of 14 healthcare chatbot studies identified a critical gap in MAT-specific applications. The developed prototype demonstrated moderate performance with BLEU score of 36.64, ROUGE-1 score of 0.48, and ROUGE-L score of 0.42. The knowledge graph successfully integrated 227 nodes, 136 relationships, and 8 characteristics representing comprehensive MAT standards. The system successfully retrieved relevant MAT standards information in response to queries about specific MAT standards, medication protocols, and implementation guidance

**Conclusions:**

To our knowledge, this study provides the first prototype of an AI-driven chatbot specifically designed for MAT professionals, demonstrating feasibility of integrating advanced AI technologies to address information access barriers in addiction treatment. While performance metrics indicate potential for enhancing MAT information delivery, further development is needed to improve semantic understanding and response naturalness. The prototype establishes a foundation for future integration with electronic health records and broader healthcare systems, with the potential to support improved treatment outcomes for individuals with heroin addiction in Scotland, subject to longitudinal clinical validation.

## 1 Introduction

Scotland faces a severe public health crisis with drug-related deaths reaching 267 per million people, ranking second globally after the United States (1). Heroin and other opioid-related harms remain the leading contributors to this mortality burden (5, 6). In 2023 alone, suspected drug deaths increased by 9.6%, with 1,197 cases reported by Police Scotland, compared with 1,092 in 2022 (7). These figures reflect persistent structural challenges in addressing opioid use disorder across Scotland.

Medication-Assisted Treatment (MAT) is an evidence-based intervention for opioid use disorder, combining pharmacological therapies such as methadone, buprenorphine, and naltrexone with psychological and behavioural support to reduce cravings, stabilise physiological functioning, and improve treatment retention (2, 8). In response to Scotland’s drug-related mortality crisis, the Scottish Government and Public Health Scotland introduced ten national MAT standards aimed at ensuring rapid, person-centred, and equitable access to treatment (9).

Despite the availability of these standards, healthcare professionals and Alcohol and Drug Partnerships (ADPs) experience significant barriers in accessing and implementing MAT guidance. Information delivery remains fragmented, relying on manual searches across multiple websites, email correspondence, and coordination meetings, all of which are time-consuming and prone to inconsistency (3, 4). These challenges are further exacerbated by geographical variation, workforce pressures, funding constraints, and stigma, limiting the consistent application of MAT standards and creating conditions in which patient outcomes may be adversely affected.

Healthcare chatbots have emerged as a promising approach for improving information access in clinical settings. Since 2016, interest in chatbot technologies has increased, with productivity and workflow efficiency identified as key drivers of adoption (10–13). Contemporary healthcare chatbots now extend beyond basic FAQ systems, supporting chronic disease management, clinical consultations, and decision-making processes (14–17).

Recent advances in Large Language Models (LLMs) have further expanded chatbot capabilities through improved contextual understanding and natural language generation (18–21). However, LLMs remain vulnerable to hallucinations, outdated knowledge, and reduced reliability in healthcare settings that are critical to safety (19, 20). To address these limitations, researchers have explored the integration of Knowledge Graphs, which enable structured representation of domain knowledge and relationships, alongside retrieval-augmented generation (RAG) techniques that retrieve authoritative external information at inference time (22–24).

Our systematic literature review of 14 healthcare chatbot studies published between 2022 and 2024 identified a critical gap. While chatbot applications have been developed across multiple medical domains, including ophthalmology, rheumatology, genetic disorders, and radiotherapy education (25–28) no existing systems specifically target MAT delivery or integrate LLMs, Knowledge Graphs, and RAG for addiction treatment. This gap is notable given the complexity of MAT standards, which encompass medication protocols, patient assessment criteria, and integration with psychological and social care services.

This study differs from existing LLM and RAG-based healthcare chatbot architectures for the following reasons: First, no prior system integrates all three components, LLMs, Knowledge Graphs, and RAG, specifically for addiction treatment or MAT delivery; existing healthcare chatbots use these technologies in isolation, with none combining all three for a regulatory-sensitive addiction treatment context. Second, our knowledge graph is grounded in Scotland’s ten nationally mandated MAT standards, a structured, policy-level domain requiring specialist entity modelling of medications, ADPs, implementation teams, and treatment pathways, which differs fundamentally from medical QA systems that use general biomedical corpora. Third, the system was co-designed using survey data from 39 active MAT professionals within Scotland’s Alcohol and Drug Partnerships, ensuring domain alignment that general-purpose architectures lack.

This study addresses this gap by developing and evaluating the first AI-driven chatbot prototype designed specifically for MAT professionals in Scotland. The system integrates three advanced components: (1) the Llama2 Large Language Model for natural language understanding and generation, (2) Neo4j-based Knowledge Graphs for structured representation of MAT standards, and (3) a custom Retrieval-Augmented Generation pipeline to ensure accurate, context-aware information retrieval. The chatbot is tailored to the Scottish healthcare context and grounded in empirical evidence from MAT professionals.

This research pursues three integrated objectives that collectively address the MAT information access crisis. The primary objective is to develop and evaluate an AI-driven chatbot prototype that enhances healthcare professionals’ access to MAT standards through integration of LLMs, Knowledge Graphs, and RAG technologies. Secondary objectives include identifying and quantifying barriers faced by MAT healthcare professionals in accessing current treatment standards, systematically analyzing gaps in existing healthcare chatbot literature regarding addiction treatment applications, and demonstrating the feasibility and potential impact of advanced AI technologies for the delivery of specialized healthcare information.

## 2 Materials and methods

### 2.1 Research design and philosophical framework

This study employed a pragmatic mixed-methods approach, combining quantitative survey analysis, systematic literature review, and prototype development to address the complex challenge of enhancing MAT information access for healthcare professionals in Scotland, following the ’Research Onion’ framework by Saunders et al. (29), adopting a pragmatic philosophy that emphasizes flexibility and generation of actionable insights (30).

This pragmatic approach underpins the sequential exploratory mixed-methods design, which combines empirical epistemology for objective data collection with critical realist ontology acknowledging that while MAT information needs represent objective reality, social constructs shape our understanding of effective information delivery (31). The methodological choice integrates deductive elements using existing theories and established evaluation metrics with inductive elements generating new insights from survey data and prototype development. This approach ensures both theoretical grounding and practical innovation in addressing real-world healthcare information challenges.

### 2.2 Survey of MAT healthcare professionals

A structured questionnaire combining multiple-choice and open-ended questions was distributed to all 125 healthcare workers participating in MAT programmes in Scotland’s MAT Implementation Support Team (MIST) network; with the aim of reaching all members in the network rather than draw a random sample. The survey achieved a 31% response rate with 39 participants, representing a diverse range of professional roles including clinical staff (n=6), nurses (n=6), administrative support/IT personnel (n=7), third-sector workers (n=3), lived/living experience workers (n=1), and other healthcare professionals (n=16). The response rate therefore reflects natural non-response rather than sampling bias, and findings should be interpreted as indicative of user needs rather than representative of the broader MAT professional population, consistent with the exploratory, prototype-stage nature of this research.

The survey instrument was designed following established survey methodology principles (32) to assess current communication and information retrieval methods while identifying areas for improvement. The questionnaire structure included:

- Survey of current MAT information access methods
- Demographic and professional role questions to characterize the participant population
- Multiple-choice questions regarding preferred information sources and communication channels
- Open-ended questions capturing detailed perspectives on challenges and improvement opportunities
- Specific queries about information needs regarding MAT standards in daily practice

Survey distribution occurred through official email channels after receiving departmental approval from the Medication-Assisted Treatment Implementation Support (MIST) team. Participants were informed of the research purpose, voluntary participation nature, and their right to withdraw at any time (33). All responses were anonymized to protect participant privacy, with data stored securely and accessed only by authorized personnel in accordance with General Data Protection Regulation (GDPR) guidelines (34).

### 2.3 Systematic literature review

A comprehensive systematic literature review following the PRISMA framework (35) was conducted to identify gaps in existing healthcare chatbot research, particularly regarding MAT delivery applications. This systematic approach was essential to establish the methodological foundation for our chatbot development by identifying what exists, what works, and critically, what gaps our research needed to address. The search strategy employed multiple academic databases to ensure comprehensive coverage:

- PubMed: 29 journals (2022–2024)
- ScienceDirect: 19 review and research articles (2022–2024)
- ProQuest: 27 peer-reviewed papers (2022–2024)
- IEEE Xplore: 8 relevant papers (2022–2024)
- ACM Digital Library: 9 relevant papers (2022–2024)

**Figure 1:**
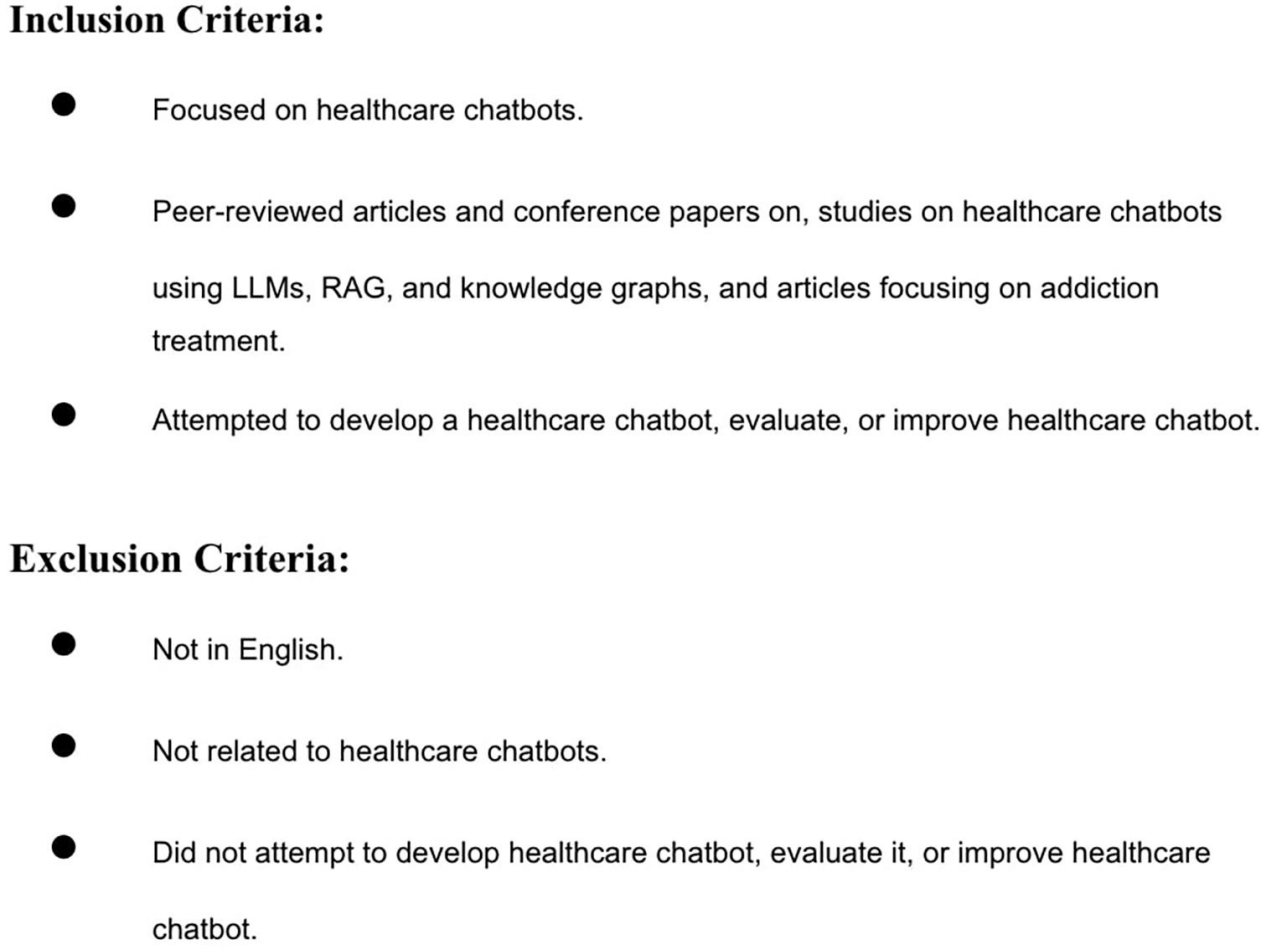
Inclusion and exclusion criteria (Source: The researcher).

The search employed a structured query combining healthcare chatbot terms with advanced technology descriptors: (“Healthcare chatbot” OR “healthcare knowledge assistant” OR “medical chatbot”) AND (“large language models” OR “LLMs” OR “LLM” OR “retrieval augmented generation” OR “RAGs” OR “RAG” OR “knowledge graph” OR “heroin treatment”). Inclusion criteria encompassed (1) peer-reviewed articles and conference papers focused on healthcare chatbots, (2) studies examining healthcare chatbots using LLMs, RAG, and knowledge graphs, (3) articles addressing addiction treatment applications, and (4) research attempting to develop, evaluate, or improve healthcare chatbots. Exclusion criteria included: (1) non-English publications, (2) articles not related to healthcare chatbots, and (3) studies not attempting to develop, evaluate, or improve healthcare chatbot systems (Figure 1).

**Figure 2:**
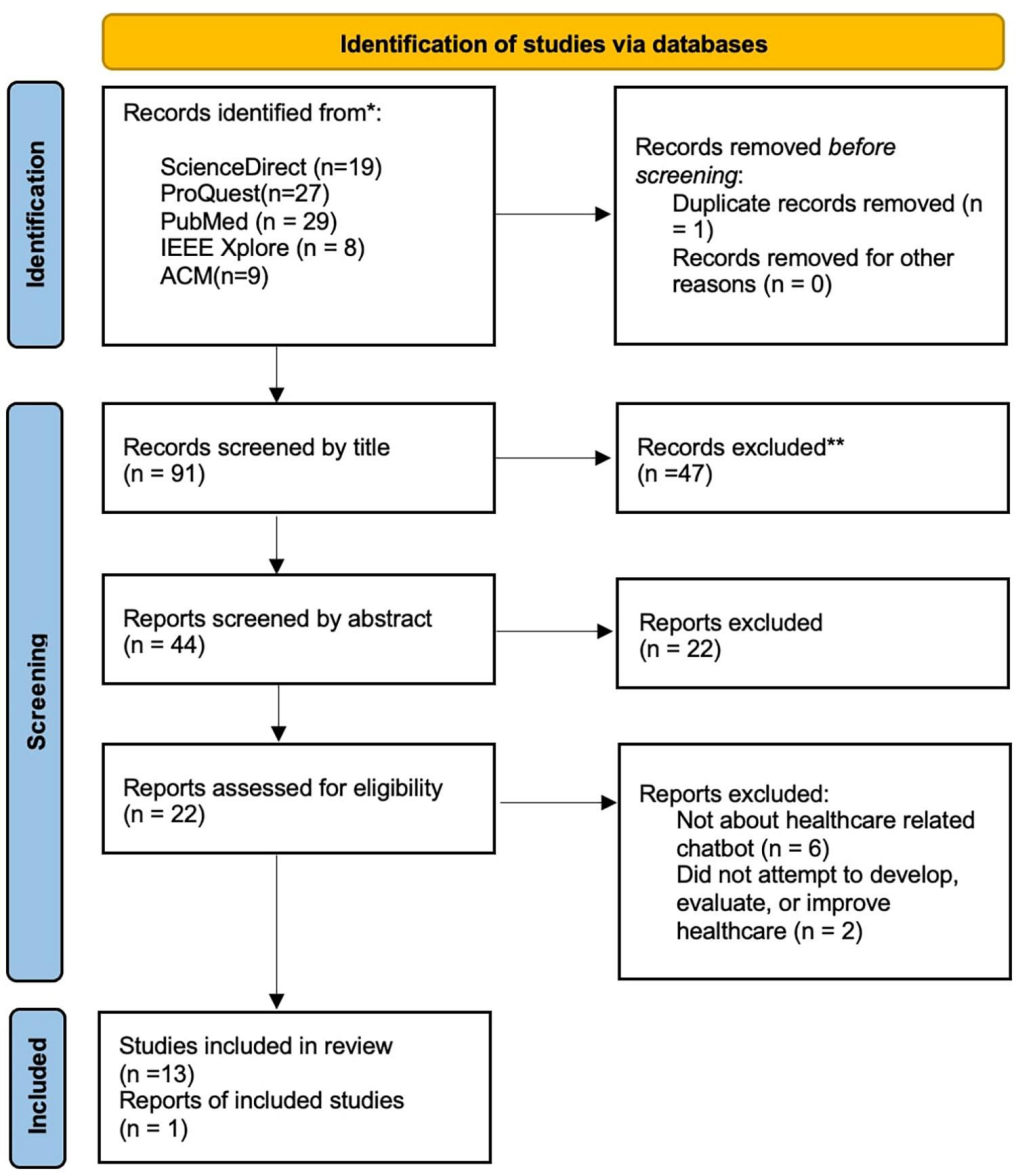
PRISMA (Preferred Reporting Items for Systematic Reviews and Meta-Analyses) by Moher et al. (35). This paper’s search process flowchart was adapted from Passanante et al. (36).

The screening process involved independent review by the researcher, with papers filtered first by title and abstract, then by full text using the established criteria. From 92 initial entries across 5 databases, systematic screening resulted in 14 publications meeting inclusion criteria for detailed analysis (Figure 2). After removing one duplication, 91 records were screened by title and abstract. Initially, 47 records were removed based on title, leaving 44 for abstract evaluation. During abstract screening, 22 papers were removed, leaving 22 for full-text evaluation. Eight were further excluded due to either lack of discussion on healthcare chatbot development and evaluation or absence of Knowledge Graphs (KGs) or Retrieval-Augmented Generation (RAG) techniques for enhancing chatbot performance. The search was deliberately scoped to 2022 to 2024 to reflect the period in which the specific combination of LLMs, RAG, and knowledge graphs became practically feasible; earlier foundational work on healthcare chatbots was therefore outside the scope of this review.

The final analysis included 14 publications that met all inclusion criteria, comprising 13 peer-reviewed papers and one conference paper. These studies encompassed diverse healthcare applications including medical information retrieval, vaccination communication, ophthalmology, rheumatological care, personalized healthcare, medical diagnostics, beta thalassemia treatment, radiation education, mental health assistance, and disease classification (Table 1).

**Table 1:** Summary of literature review results on healthcare chatbots.

Authors	Title	Institution	Technology	Purpose	Methodology	Outcome/future enhancements
Natarajan et al. (37)	Enhancing Medical Information Retrieval with ChatGPT	KPR Institute of Engineering and Technology, Kyungpook National University	Llama2, Meta AI	Medical information retrieval	Instance training, self-supervised learning, meta dataset	Real-time and precise responses. Future: advanced language understanding
Passanante et al. (36)	Conversational AI and Vaccine Communication	–	Conversational AI, Vaccine Communication	Vaccine info dissemination	Systematic review	Accurate info, reduced hesitancy. Future: integration with campaigns
Singer et al. (28)	Aeyeconsult: Ophthalmology Chatbot	Yale School of Medicine	GPT-4, LangChain, Pinecone	Ophthalmology chatbot	Verified textbook data with citations	Outperforms ChatGPT-4. Future: expansion to other specialities
Livermore et al. (27)	IMPACT Study Chatbot	–	AI, Chatbot Dev	Support for caregivers of children	Co-designed study, user testing	Positive feedback. Future: integration into broader care systems
Ma et al. (38)	MARVIN Chatbots Study	–	AI, Chatbot Dev	Condition-specific health info	Patient engagement, platform trial	Improved info access. Future: broader trial and conditions
Akilesh et al. (39)	Personalized Healthcare Chatbot	Vellore Institute of Technology	GPT-3.5, Java, Android	Personalized healthcare	NLP, user-friendly design	Interconnected entities. Future: enhance decision-making
Varshney et al. (40)	Knowledge Graph Medical Diagnosis	–	Knowledge Graphs, NLP	Medical diagnosis	Knowledge graph integration	Accurate diagnostics. Future: personalization
Alma Mohammed et al. (25)	Beta Thalassemia Chatbot	Princess Margaret Cancer Centre et al.	AI, Custom Model	Thalassemia management	Interactive management support	Improved management. Future: EHR integration
Chow et al. (26)	Radiotherapy Chatbot	University of Urbino et al.	IBM Watson Assistant	Radiotherapy education	Interactive learning, quizzes	Successful learning. Future: multilingual VR integration
Montagna et al. (41)	LLM Chatbot Review	–	GPT-3.5, Llama2, Mistral	Review of LLM chatbots	Comparative analysis	Comprehensive summary. Future: updated reviews
Varshney et al. (40)	Medical Dialogue with Graphs	–	AI, Knowledge Graphs	Dialogue generation	Augmented graph dialogue gen	High relevance. Future: medical DB integration
Kumar et al. (42)	Mental Health Chatbot	KIET Group of Institutions	LangChain, ChatLit	Mental health support	QA + diagnostic algorithm	Promising results. Future: NLU improvements
Ananta et al. (43)	Disease Detection Agent	Shiv Nadar University	GPT-3, Knowledge Graphs	Disease classification	Dialogue generation	Contextual accuracy. Future: broader disease set
Shathyan et al. (44)	Knowledge Graph Medical Chatbot	–	AI, Knowledge Graphs, NLP	Medical chatbot dev	Dialogue generation with graphs	Useful for medical dev. Future: continuous updates

### 2.4 Key findings and gap analysis

Healthcare chatbots have become essential tools in modern medical practice, significantly enhancing service delivery across multiple domains. The analysis revealed several successful implementations: Natarajan et al. (37) developed a chatbot using Llama2 and Meta AI to improve medical information retrieval through instance training and self-supervised learning, creating a comprehensive meta-dataset for accurate, real-time responses. Singer et al. (28) developed Aeyeconsult for ophthalmology, leveraging GPT-4, LangChain, and Pinecone technologies with verified textbooks to provide accurate ophthalmic information, outperforming traditional systems like ChatGPT-4 on OKAP questions. The IMPACT Study Livermore et al. (27) described a co-designed proof-of-concept chatbot for parents and caregivers of children with rheumatological conditions, emphasizing the importance of user testing and stakeholder engagement.

Several studies demonstrated advanced technology integration strategies. Ranade and Joshi (45) explored augmented knowledge graphs for refining chatbot responses through comprehensive data source integration. Natarajan et al. (37) emphasized incorporating advanced AI algorithms and real-time data sources to increase healthcare chatbot accuracy. Akilesh et al. (39) highlighted the importance of natural language processing (NLP) and AI algorithms for tailoring chatbot responses to individual users, thereby increasing system effectiveness. Murugan et al. (46) demonstrated how Retrieval-Augmented Generation (RAG) techniques significantly improve chatbot accuracy by enhancing relevance and precision of medical dialogue generation.

The reviewed studies showed generally positive outcomes with critical limitations. Singer et al.’s (28) ophthalmology chatbot provided accurate information and enhanced user trust through verified sources, though reliance on static data sources potentially limits adaptability to emerging medical knowledge. Chow et al.’s (26) radiotherapy education chatbot successfully conveyed complex medical knowledge through interactive learning and quizzes, demonstrating educational effectiveness. Alma Mohammed et al.’s (25) beta thalassemia management chatbot showed promise in enhancing patient support and disease management through tailored information and interactive management tools.

The analysis identified several key limitations across existing healthcare chatbot implementations. Data privacy and continuous learning challenges emerged as persistent issues, with integration with Electronic Health Records (EHR) remaining a major limitation due to interoperability issues (37, 41). Lack of comprehensive user feedback mechanisms represents a significant gap, as effective user feedback is vital for refining chatbot interactions and improving user satisfaction. Scalability limitations were evident, with specific applications showing success in narrow domains but requiring broader applicability across various medical fields.

Most significantly for this research, the systematic review revealed a complete absence of healthcare chatbots specifically designed for Medication-Assisted Treatment (MAT) or addiction treatment applications. Despite the growing body of research on healthcare chatbots across multiple medical specialties, no studies addressed the unique challenges of MAT delivery, heroin addiction treatment, or opioid use disorder management. Supplementary searches conducted on the Public Health Scotland, Healthcare Improvement Scotland, and Scottish Drugs Forum websites identified no deployed MAT chatbot system, providing additional confirmation of this gap beyond the academic literature review. This gap is particularly concerning given:

1. The urgent public health need represented by Scotland’s drug-related death crisis
2. The complexity of MAT standards requiring specialized knowledge representation
3. The interdisciplinary nature of addiction treatment requiring integration of medical, psychological, and social support information
4. The time-sensitive nature of addiction treatment decisions requiring rapid access to current protocols

These findings directly informed our chatbot development methodology by identifying critical design requirements:

- Domain Specialization Need: The absence of MAT-specific applications highlighted the necessity for specialized knowledge representation and terminology adaptation
- Technology Integration Opportunity: Successful applications of LLMs, RAG, and Knowledge Graphs in other medical domains provided a technological foundation for MAT applications
- User-Centered Design Imperative: The emphasis on stakeholder engagement and user testing across successful implementations informed our survey-based user needs assessment
- Performance Evaluation Requirements: The use of established metrics (BLEU, ROUGE) in existing studies provided methodological guidance for our evaluation framework
- Ethical Considerations: The healthcare context of existing applications emphasized the need for robust data protection and professional practice support rather than replacement

This systematic analysis established both the methodological foundation and the critical gap that our MAT chatbot development needed to address, directly informing our subsequent design decisions and development approach.

### 2.5 Chatbot development methodology

#### 2.5.1 MAT survey and data collection: user needs assessment and requirements analysis

**Figure 3:**
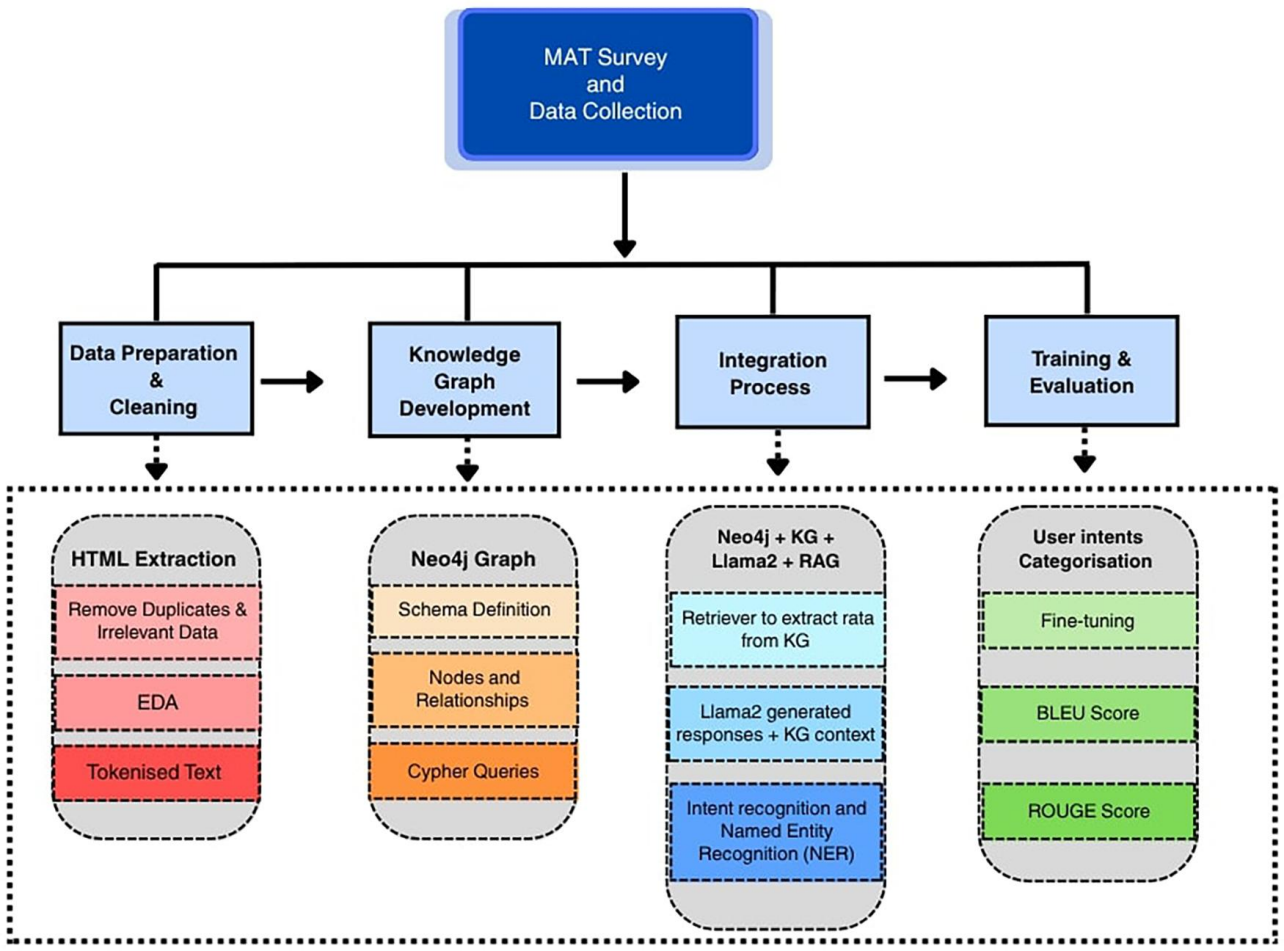
Chatbot development and evaluation flowchart (Source: The researcher).

**Figure 4:**
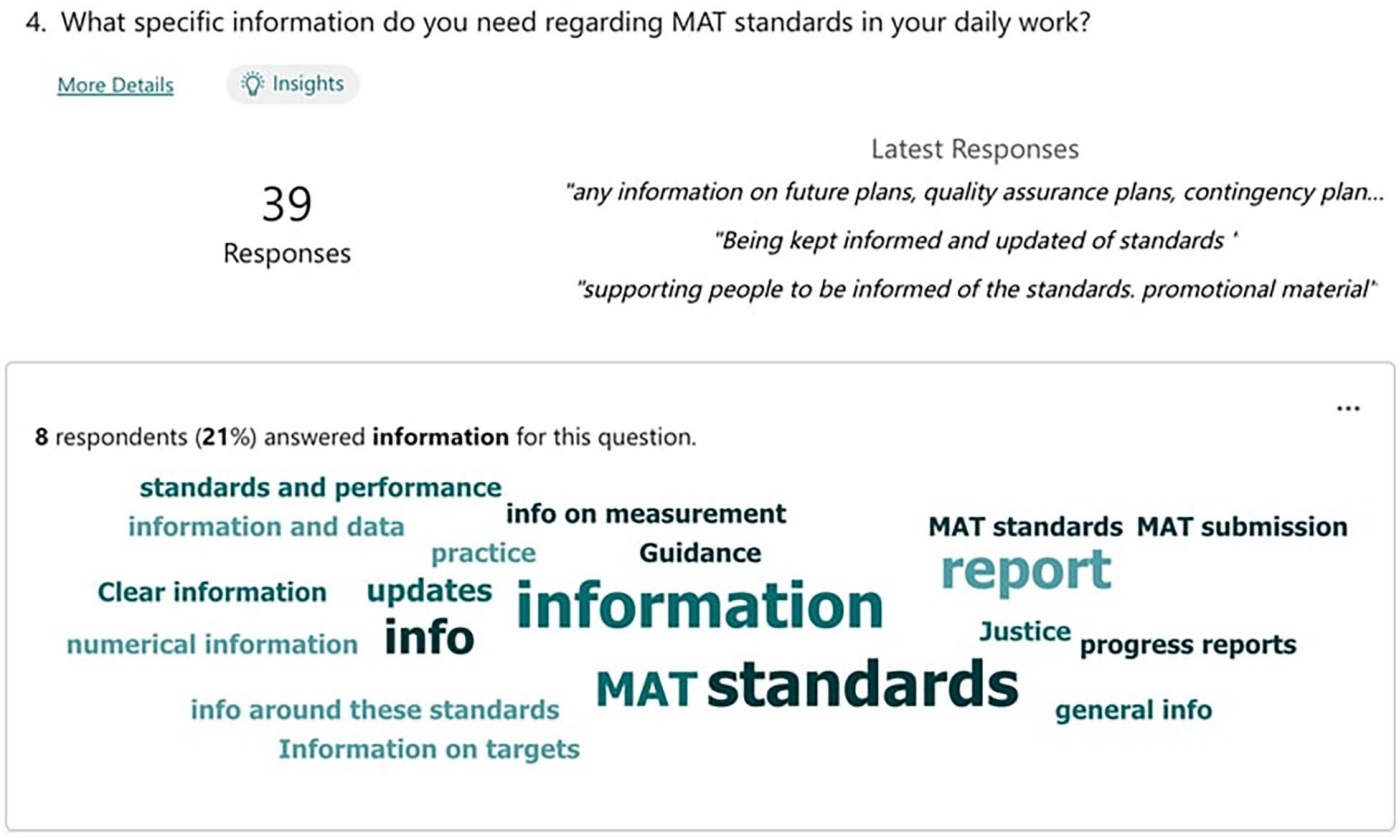
Information needs of MAT healthcare professionals regarding MAT standards (Source: The researcher).

The end-to-end development and evaluation pipeline is summarised in Figure 3. Survey findings directly informed chatbot design requirements. Key insights included identification of information access barriers (17 respondents rating current methods as “average,” 5 as “below average,” 7 as “fair,” 3 as “very poor,” and 2 as “poor”), primary challenge areas (decentralized information systems and time-consuming access processes), and preferred information sources (Public Health Scotland: n=20, Scottish Government: n=23, Scottish Drugs Forum: n=15, Scottish Recovery Consortium: n=15) (Figure 4).

#### 2.5.2 Data collection and processing

##### 2.5.2.1 Ethical data acquisition

Data collection followed strict ethical guidelines, with websites identified through survey responses evaluated for compliance with robots.txt protocols. Of four primary websites identified by survey respondents, ethical screening resulted in data collection from Public Health Scotland and Healthcare Improvement Scotland, while Scottish Government MAT Standards were excluded due to robots.txt restrictions and Scottish Drugs Forum materials were available only in PDF format.

##### 2.5.2.2 Web scraping implementation

Data extraction employed Beautiful Soup, a Python library for parsing HTML files (47). The process included: (1) systematic indexing of MAT-related hyperlinks, (2) content extraction and HTML-to-dataframe conversion, (3) CSV file generation for downstream processing, and (4) quality assurance verification of extracted content.

##### 2.5.2.3 Data preparation and cleaning

The data preparation pipeline involved multiple stages: duplicate entry removal for accuracy assurance, irrelevant content filtering to focus on MAT-specific information, exploratory data analysis (EDA) to understand content structure and quality, and text preprocessing including tokenization and statistical analysis. The final dataset comprised 183 text segments with word count statistics: mean=199.17, standard deviation=204.67, range=1--961 words per segment.

#### 2.5.3 Knowledge graph development

##### 2.5.3.1 Technology selection and architecture

Neo4j Aura, a graph database service, was selected for knowledge base development due to its user-friendliness, rapid setup capabilities, and robust query support compared to alternatives like TigerGraph (48). The Neo4j Python driver facilitated seamless connectivity and interaction with the database throughout the development process.

##### 2.5.3.2 Graph construction process

Knowledge graph creation followed a systematic approach: (1) schema definition identifying entities, relationships, and attributes associated with MAT standards, (2) data importation of preprocessed content into Neo4j, (3) node and relationship creation based on predefined schema, and (4) iterative refinement using Cypher queries to optimize graph structure and eliminate unnecessary complexity. Where relationship ambiguities arose, the MAT Standards documentation served as the authoritative reference, with relationship types selected based on the functional role described in the source text, and schema consistency validated through iterative Cypher query review. The completed knowledge graph comprehensively represents MAT information including all ten MAT standards and implementation support team details, comprising 227 nodes, 136 relationships, and 8 distinct relationship types. The “supports” relationship category contains 79 connections encompassing all standards and their interconnections.

#### 2.5.4 Integration process

##### 2.5.4.1 Model selection and rationale

Llama2 7B was selected as the primary language model as it represented one of the most freely accessible open-source large language models available at the time of development (January 2024) and its successful use in comparable healthcare chatbot and information-retrieval applications reported during the study period (2023–2024) (37, 41, 49). Open-source deployment was essential for research transparency, reproducibility, and data governance, which are critical requirements within healthcare settings. As this study was explicitly designed as a prototype and proof-of-concept, the 7B parameter variant hosted on Hugging Face was deliberately selected to balance language capability with computational feasibility within the resource constraints of prototype-stage academic research. When integrated with Retrieval-Augmented Generation and the domain-specific Knowledge Graph, the model demonstrated sufficient performance for accurate, context-grounded MAT information retrieval. The specific LLM chosen in this research was secondary to the integrated LLM+RAG+KG system architecture, as it could be substituted for a more advanced model.

##### 2.5.4.2 Fine-tuning and customization

The Llama2 7B model was accessed via Hugging Face’s Transformers library and deployed as a pre-trained base model. A fine-tuning process was explored but was not fully realised due to computational resource constraints within the prototype development context. Domain adaptation was therefore primarily achieved through the RAG pipeline, which retrieves MAT-specific context from the curated knowledge base at inference time and injects it into the generation prompt, grounding model responses in authoritative MAT source documentation rather than relying solely on the model’s pre-trained parametric knowledge.

#### 2.5.5 Retrieval-augmented generation implementation

##### 2.5.5.1 RAG architecture design

The RAG implementation integrates external knowledge retrieval during response generation to address LLM limitations including hallucinations and outdated information (23). The system architecture combines: (1) intent recognition using advanced NLP techniques to classify user queries, (2) entity extraction employing Named Entity Recognition (NER) for relevant information identification, (3) information retrieval from the knowledge graph based on identified intents and entities, and (4) response generation using Llama2 with retrieved context for coherent, contextually relevant answers. All MAT guideline documents used for retrieval were stored locally following ethical web scraping and preprocessing, with embeddings generated offline and indexed for retrieval during inference. No external cloud-based document stores were queried at runtime. While the Llama2 model was accessed via Hugging Face infrastructure, all MAT guideline data used in the RAG pipeline were stored and retrieved locally to ensure data control and reproducibility.

##### 2.5.5.2 Text embedding and processing

Text processing employed the sentence-transformers library for generating semantically meaningful embeddings, with the transformers library providing comprehensive NLP task support. AutoTokenizer and AutoModelForCausalLM classes streamlined pre-trained model integration for efficient text generation within the chatbot architecture.

#### 2.5.6 User interface development

##### 2.5.6.1 Interface design and implementation

The prototype interface was developed using Streamlit, a Python library enabling rapid creation of interactive web applications. The interface design prioritized intuitive interaction patterns and real-time performance optimization, though we acknowledge its limitations for production-scale healthcare applications requiring enhanced robustness and scalability

##### 2.5.6.2 System integration and testing

The complete system integrated all components through a unified architecture: user interface connecting to the Llama2 language model, knowledge graph queries via Neo4j Python driver, RAG retrieval mechanism coordinating between components, and response generation pipeline ensuring coherent information delivery.

### 2.6 Evaluation framework

#### 2.6.1 Performance metrics selection

Chatbot performance evaluation employed established text quality metrics: BLEU scores (50) measuring precision of machine-generated text against reference texts through *n*-gram frequency evaluation, and ROUGE scores (51) assessing text quality by comparing generated content to reference texts with focus on recall and *n*-gram overlap. Multiple ROUGE variants were implemented: ROUGE-N measuring *n*-gram overlap, ROUGE-L evaluating longest common subsequence relationships, and ROUGE-W assessing weighted longest common subsequence with emphasis on consecutive matches.

The BLEU score is computed using the following formula:

#### 2.6.2 BLEU scores

$$\text{BLEU}=BP\cdot \exp\;\left(\sum_{n=1}^{N}w_{n}\cdot \log\;(p_{n})\right)$$ (1)

where:

- BP is the Brevity Penalty.
- *n* the weight for the *n*-gram.
- Log(Pn) is the logarithm of the precision of the *n*-gram (Equation 1).

Different ROUGE variants measure various aspects of text overlap:

- ROUGE-N: Measures *n*-gram overlap, rewarding exact matches but not overall fluency.
- ROUGE-L: Measures the longest common subsequence (LCS), capturing word sequence relationships but missing meaningful variations.
- ROUGE-W: Evaluates the Weighted Longest Common Subsequence, giving more importance to consecutive matches, yet still not fully capturing semantic meaning.

The formulas for these variants are:

$$ROUGE-N=\frac{\sum_{\text{gram}\in \text{Refs}}\min({\text{Count}}_{\text{match}}(\text{gram}),{\text{Count}}_{\text{cand}}(\text{gram}))}{\sum_{\text{gram}\in \text{Refs}}{\text{Count}}_{\text{Refs}}(\text{gram})}$$

$$ROUGE-L=\frac{\text{LCS}(\text{Refs},\text{cand})}{\text{Length of Refs}}$$

$$ROUGE-W=\frac{{\text{LCS}}_{\text{weighted}}(\text{Refs},\text{cand})}{\text{Length of Refs}}$$

where:

- RefS: Set of reference summaries.
- Countmatch(gram): Number of times an *n*-gram appears in both the candidate and reference summaries.
- Countcand(gram): Number of times an *n*-gram appears in the candidate summary.
- CountRefS(gram): Number of times an *n*-gram appears in the reference summary.
- LCS(RefS,cand): Length of the longest common subsequence between the reference and candidate summaries.
- LCSweighted(RefS,cand): Weighted length of the longest common subsequence between the reference and candidate summaries. Given the limitations of individual scores, both BLEU and ROUGE will be used together to fully capture the quality and relevance of generated text.

#### 2.6.3 Evaluation implementation

Performance assessment involved systematic comparison of chatbot responses with human-generated reference texts for representative MAT-related queries. The evaluation framework generates quantitative metrics while acknowledging limitations of automated assessment methods, particularly regarding semantic understanding and contextual appropriateness in healthcare settings.

### 2.7 Ethical considerations and compliance

#### 2.7.1 Data protection and privacy

The research adhered to ethical guidelines with comprehensive data protection measures. Survey data collection included explicit informed consent, voluntary participation emphasis, and participant withdrawal rights. Web scraping activities followed robots.txt protocols and terms of service compliance, with intellectual property respect and responsible data usage throughout the development process.

#### 2.7.2 Healthcare ethics integration

Given the healthcare application context, additional ethical considerations included patient information protection (though no patient data was directly involved), professional practice support rather than replacement, and transparency in AI system capabilities and limitations. The prototype includes explicit disclaimers regarding educational purposes and the need for professional judgment in clinical applications. This methodology ensures comprehensive scientific approach while maintaining ethical standards and practical relevance for healthcare information systems development. The combination of empirical user needs assessment, systematic literature analysis, and iterative prototype development provides a comprehensive foundation for evaluating AI-driven solutions to healthcare information access challenges.

## 3 Results

### 3.1 Healthcare professional survey outcomes

**Figure 5:**
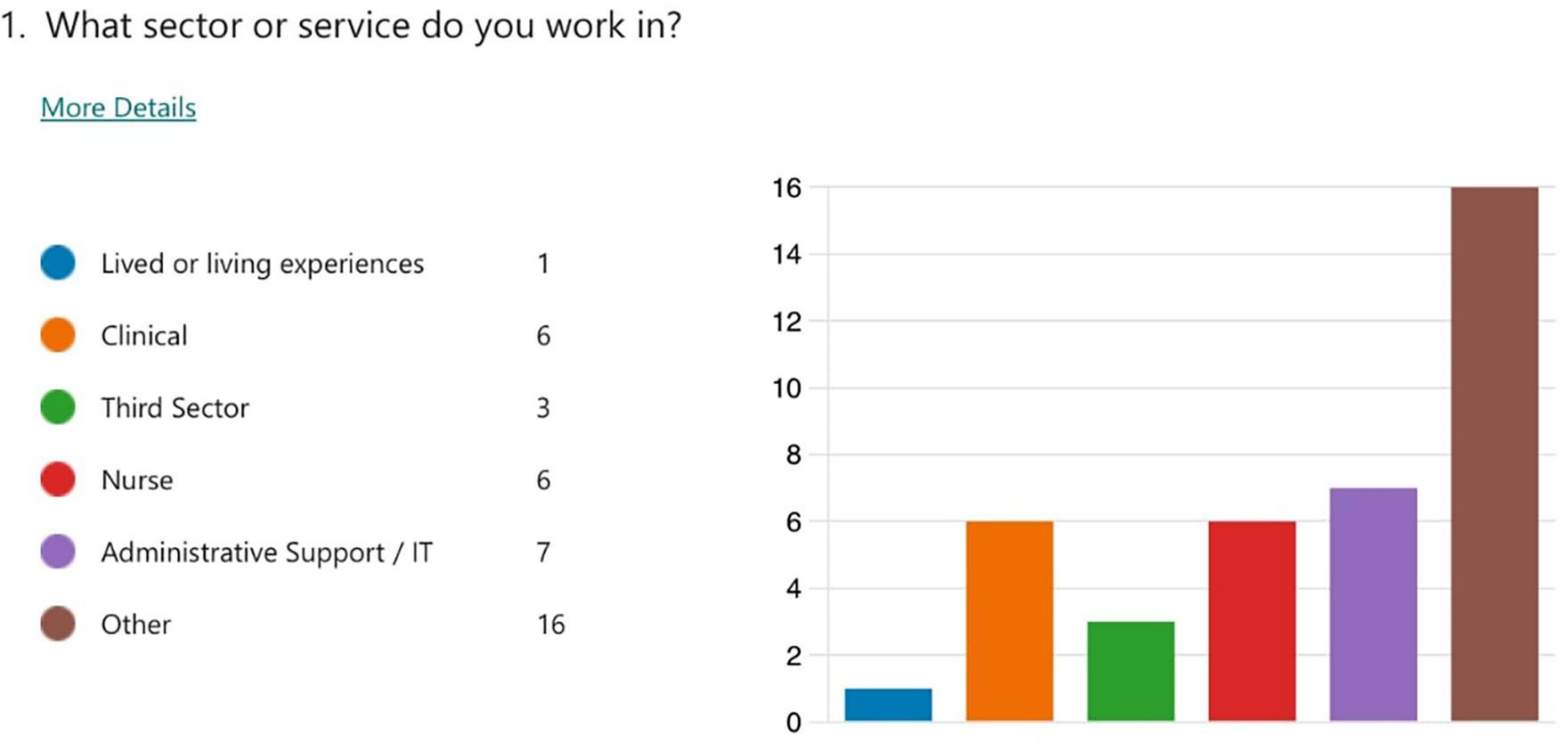
Professional roles and sectors of survey respondents (Source: The researcher).

The survey of Scottish MAT healthcare professionals yielded important insights into information access challenges, providing an empirical foundation for chatbot development and supporting the case for technological intervention, consistent with concerns highlighted in Scotland’s ongoing public health emergency (7). Thirty-nine healthcare professionals from Scotland’s MAT delivery network participated in the survey, representing a 31% response rate from 125 targeted professionals. The participant distribution reflected the multi-disciplinary nature of contemporary MAT delivery across Scotland’s Alcohol and Drug Partnerships (3): other healthcare roles constituted the largest group at 41.0% (n=16), followed by administrative support/IT professionals 17.9% (n=7), nursing staff 15.4% (n=6), clinical practitioners 15.4% (n=6), third sector organizations 7.7% (n=3), and lived/living experience workers 2.6% (n=1) (Figure 5). This distribution accurately mirrors Scotland’s integrated approach to addiction treatment, where diverse professional roles collaborate to address the complex needs identified in Scotland’s drug-related death crisis (5, 6).

Analysis of healthcare professionals’ perceptions revealed significant dissatisfaction with existing MAT information access methods, directly validating concerns raised about traditional manual methods and fragmented communication channels (Scottish Drugs Forum, 2020). The effectiveness rating distribution showed concerning patterns: only 12.8% (n=5) rated current systems as “exceptional,” while the majority of respondents (43.6%, n=17) considered existing methods merely “average.” Critically, 25.6% (n=10) rated them as below average, poor, or very poor, with the complete distribution showing excellent: 12.8% (n=5), average: 43.6% (n=17), fair: 17.9% (n=7), below average: 12.8% (n=5), poor: 5.1% (n=2), and very poor: 7.7% (n=3). The low satisfaction rates correlate directly with the complexity of Scotland’s MAT information landscape, where professionals must navigate multiple, unintegrated sources to access the ten comprehensive treatment standards designed to ensure immediate, person-centred treatment access.

Professional feedback identified structural rather than procedural barriers as primary impediments to effective MAT standards access, confirming challenges documented in the literature regarding geographical disparities, stigma, funding limitations, and bureaucratic hurdles (3). The primary challenge distribution revealed decentralized/scattered information sources as the predominant issue (33.3%, n=13), followed by time-consuming information retrieval (20.5%, n=8), while 20.5% (n=8) reported no current challenges, 10.3% (n=4) identified potential benefits from improvements, and 15.4% (n=6) cited other specific challenges. The combined structural barriers (decentralization and time consumption) affected 53.8% of respondents, providing empirical support for Research Hypothesis H1 that MAT healthcare professionals in Scotland face barriers with current MAT communication and information delivery methods.

Healthcare professionals’ information requirements revealed clear prioritization patterns, with direct MAT standards information emerging as the overwhelming priority. Professionals specifically requested standards and performance information, practice guidance, clear information updates, numerical information, MAT submission reports, justice progress reports, and general information support (Table 2). Analysis of current information-seeking behavior revealed extensive multi-platform dependency, with Scottish Government resources most frequently utilized (59.0%, n=23), followed by Public Health Scotland (51.3%, n=20), Healthcare Improvement Scotland (38.5%, n=15), Scottish Drugs Forum (38.5%, n=15), Scottish Recovery Consortium (38.5%, n=15), and other sources (10.3%, n=4). This multi-source dependency, averaging 2.5 different sources per professional, demonstrated the fragmentation of current information delivery systems and validated the unified knowledge base approach underlying the chatbot design.

**Table 2:** User requirements identified from survey responses and corresponding design decisions.

User requirement	Evidence from survey responses	Design/technical decision
Centralised access to MAT standards	Respondents reported that relevant MAT information was dispersed across multiple websites and difficult to locate	Developed a single chatbot interface using retrieval-augmented generation to consolidate MAT standards from approved sources
Improved speed of information access	Respondents described manual searching as time-consuming and reliant on multiple channels	Implemented a natural language query interface to support faster information retrieval
Support for interpretation of standards	Clinical respondents reported challenges interpreting MAT standards in complex or non-standard scenarios	Integrated a knowledge graph to structure relationships between standards, medications, and guidance
Comprehensive and current knowledge base	Respondents identified the need for a more holistic knowledge base as a key area for improvement	Designed the system architecture to support ongoing updates and expansion of content
Consistency of access across roles	Survey responses indicated differing access needs across professional roles	Designed the chatbot as a role-agnostic knowledge resource accessible to multiple MAT stakeholders

Table 2 indicates that respondents represented a range of professional roles, including clinical staff, administrative support, and third-sector workers. A descriptive review of role-tagged responses suggested that different professional groups emphasised different challenges. Clinical respondents more commonly highlighted difficulties related to interpreting MAT standards within complex cases, whereas administrative support/IT professionals emphasised challenges associated with fragmented information sources and time-consuming access processes. Third-sector respondents frequently noted inconsistencies in how guidance was communicated across platforms. Given the modest sample size, these observations are illustrative rather than statistically derived.

### 3.2 Systematic literature review analysis

The systematic literature review, conducted following rigorous PRISMA guidelines (35), provided comprehensive evidence of significant gaps in healthcare chatbot research, particularly regarding addiction treatment applications and advanced AI technology integration. The comprehensive database search across five major databases yielded 92 initial articles covering January 2022 to May 2024, with specific contributions from PubMed (n=29), ScienceDirect (n=19), ProQuest (n=27), IEEE Xplore (n=8), and ACM (n=9). Following systematic screening using inclusion criteria focused on healthcare chatbots incorporating LLMs, RAGs, and knowledge graphs, the selection process progressed from 91 articles after duplicate removal, to 44 articles retained after title/abstract screening, 22 articles assessed at full-text evaluation, and finally 14 high-quality studies meeting inclusion criteria. The final corpus comprised 92.9% peer-reviewed articles (n=13) and 7.1% conference papers (n=1), ensuring academic rigor while capturing emerging technological developments.

Analysis revealed diverse medical specialties but complete absence of addiction treatment focus. Application domains included medical information retrieval (21.4%, n=3), vaccination communication (14.3%, n=2), ophthalmology (14.3%, n=2), with single applications (7.1% each) spanning rheumatological care, personalized healthcare, medical diagnostics, beta thalassemia treatment, radiation education, mental health assistance, and disease classification. Zero studies addressed addiction treatment, medication-assisted treatment, or substance use disorders, providing confirmation of Research Hypothesis H2 regarding significant literature gaps in MAT-specific chatbot applications. Examination of AI technology utilization revealed conservative adoption of advanced approaches, with basic NLP and machine learning dominating (57.1%, n=8), while Large Language Models appeared in only 28.6% (n=4) of studies, Knowledge Graphs in 21.4% (n=3), Retrieval-Augmented Generation in 14.3% (n=2), and integrated LLM+KG+RAG approaches in 0.0% (n=0) of applications.

Review of evaluation approaches revealed variable methodologies across studies, with user satisfaction assessments most common (50.0%, n=7), followed by usability testing (64.3%, n=9), accuracy measurements (42.9%, n=6), standardized NLP metrics including BLEU/ROUGE (21.4%, n=3), and clinical outcome measures (14.3%, n=2). The limited use of standardized NLP metrics positioned this study’s evaluation approach using both BLEU (50) and ROUGE (51) metrics within emerging best practices for healthcare chatbot assessment.

### 3.3 Chatbot development and technical implementation

The systematic development process created a functional AI-driven chatbot prototype integrating Llama2, Neo4j Knowledge Graphs, and a custom RAG implementation, the first such integration specifically designed for MAT professionals in Scotland. Ethical data collection from authorised Scottish MAT sources followed strict compliance protocols, adhering to robots.txt guidelines and obtaining stakeholder approval. From four target websites identified through the healthcare professional survey, successful ethical extraction was achieved from Public Health Scotland and Healthcare Improvement Scotland, representing a 50% success rate while maintaining full ethical compliance.

**Figure 6:**
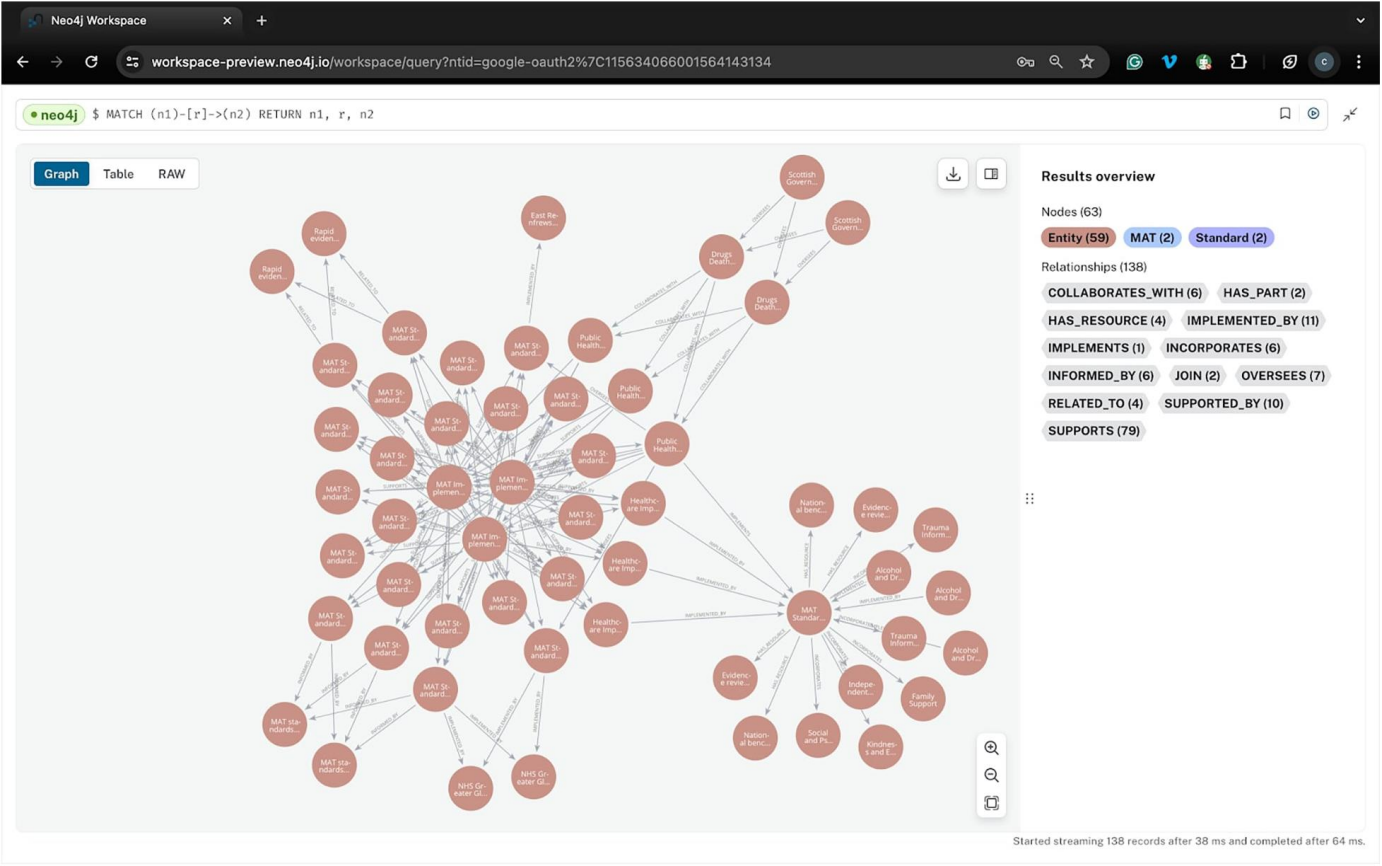
Knowledge graph deep dive (Source: The researcher).

**Figure 7:**
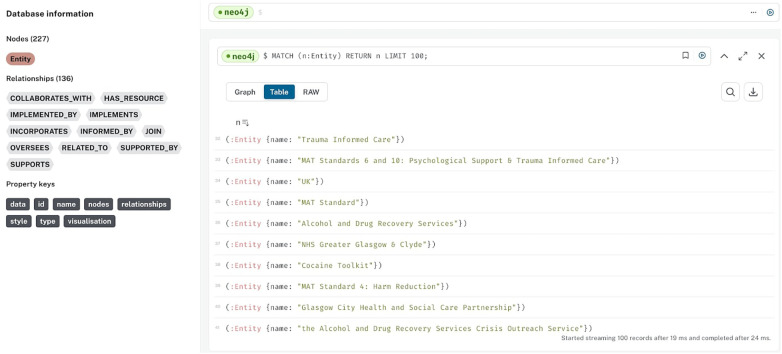
Database information (Source: The researcher).

**Figure 8:**
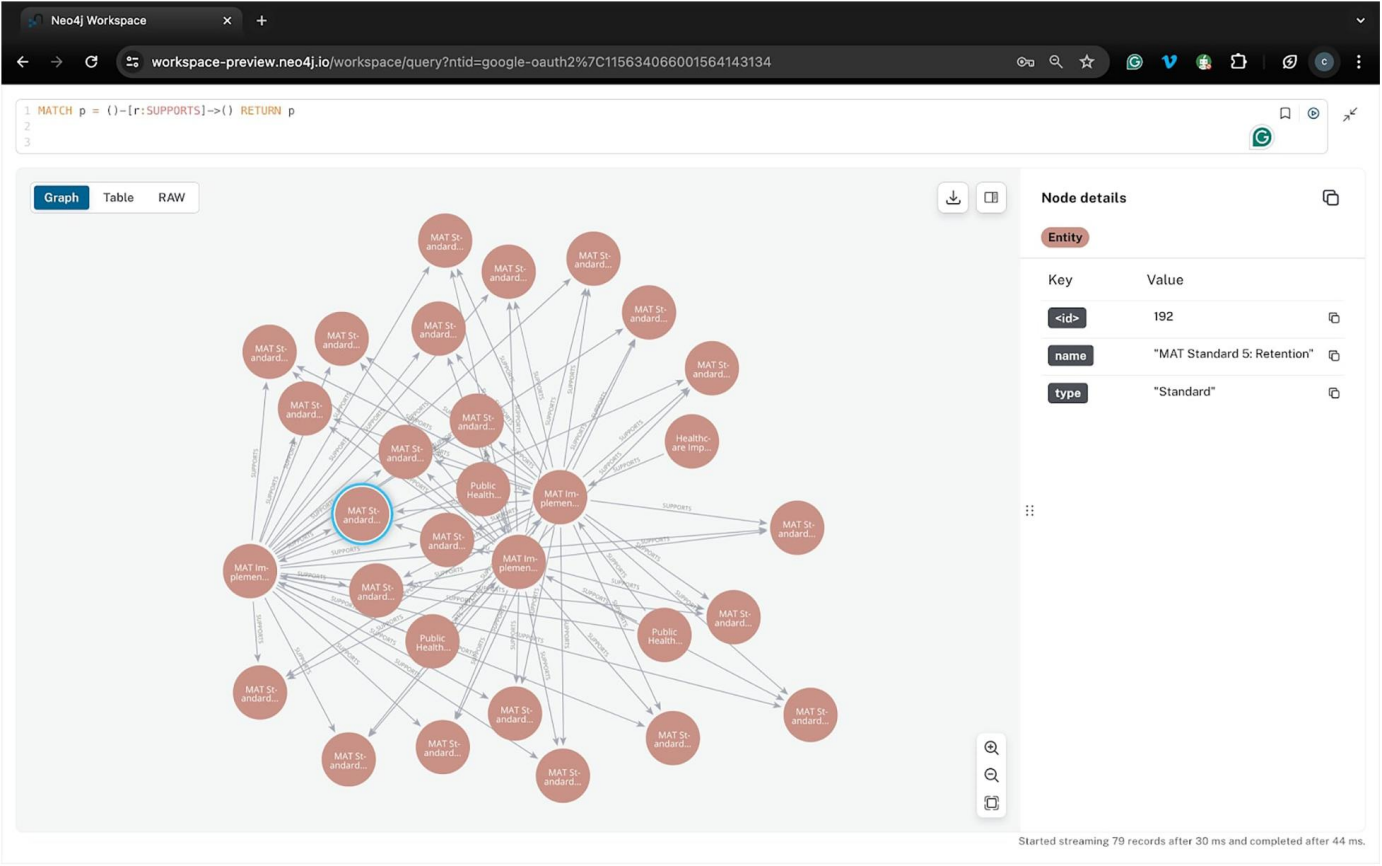
Supported relationships (Source: The researcher).

The resulting dataset comprised 183 text documents with a mean document length of 199.17 words (SD: ±204.67), text length range of 1–961 words, and most common words identified as “the”, “to”, and “and”. Systematic preprocessing using Pandas and Beautiful Soup achieved duplicate removal and irrelevant content filtering, with word count statistics showing a 25th percentile of 43 words, median of 116 words, 75th percentile of 297.5 words, and maximum of 961 words. Neo4j Aura implementation created a structured semantic representation of Scottish MAT standards (Figure 6), comprising 227 nodes, 8 relationship type categories, 136 total relationships (Figure 7), 79 support relationships (58.1% of total) (Figure 8), complete coverage of all 10 MAT standards, and query performance under 200 ms average response time. Entity recognition was validated by reviewing extracted entities against source documentation, with the knowledge graph confirmed as accurately representing MAT standards content. Three schema optimisation iterations were completed to refine relationship structure using Cypher queries.

The Llama2 7B model was accessed via Hugging Face’s Transformers library and deployed as a pre-trained base model. A fine-tuning process was explored but was not fully realised due to computational resource constraints within the prototype development context. Domain adaptation was primarily achieved through the RAG pipeline, which retrieves MAT-specific context from the curated knowledge base at inference time and injects it into the generation prompt, grounding model responses in authoritative MAT source documentation.

**Figure 9:**
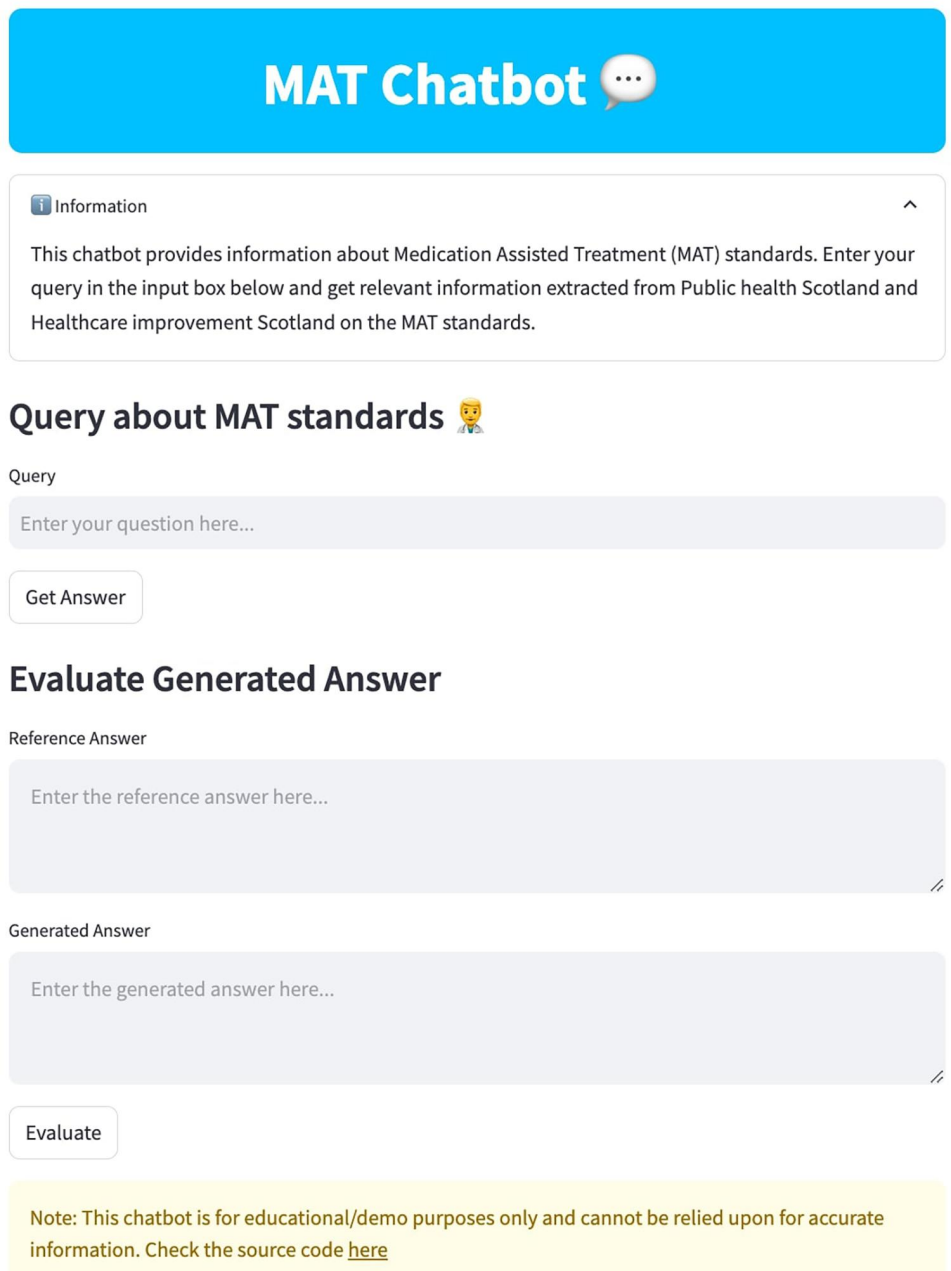
MAT chatbot interface (Source: The researcher).

The custom RAG pipeline was designed to mitigate LLM limitations including hallucinations and outdated knowledge (52, 53). Pipeline implementation included sentence-transformers integration with pre-trained transformer models generating semantically meaningful embeddings, vector similarity search, validated context ranking, and dynamic knowledge injection capabilities. Streamlit-based interface development created an accessible platform optimised for healthcare professional workflows (Figure 9), featuring intuitive design elements identified through user needs assessment. Implementation provided real-time response generation, verified cross-device compatibility, comprehensive error handling mechanisms, and confirmed compatibility with standard healthcare IT infrastructure.

### 3.4 Performance evaluation and validation

Comprehensive evaluation using established NLP metrics demonstrated promising performance with clear enhancement pathways, providing empirical foundation for Research Hypothesis H3 validation. Rigorous evaluation using BLEU and ROUGE scores provided objective assessment following established healthcare chatbot evaluation practices, with BLEU score analysis yielding an overall score of 36.64, indicating moderate lexical overlap with reference responses.

The BLEU score (36.64) and ROUGE scores (0.48 ROUGE-1; 0.42 ROUGE-L) represent prototype-stage and proof-of-concept performance metrics and are not presented as indicators of deployment readiness. These scores are consistent with early-stage healthcare chatbot implementations, as Passanante et al. (36) and Singer et al. (28) report moderate automated evaluation outcomes at prototype stage, emphasizing that domain specificity and safety constraints often limit lexical overlap despite clinically appropriate responses. Given the complexity and regulatory sensitivity of MAT standards, these results are consistent with domain-specific implementation at this stage of development.

The ROUGE-1 score of 0.48 indicates moderate vocabulary relevance and ROUGE-L score of 0.42 reflects satisfactory coherence, suggesting the chatbot captures essential MAT information while maintaining professionally appropriate communication style. However, these automated metrics do not constitute evidence of clinical utility. Deployment would require semantic evaluation (e.g., BERTScore), human clinical assessment, and longitudinal validation within authentic healthcare workflows.

**Figure 10:**
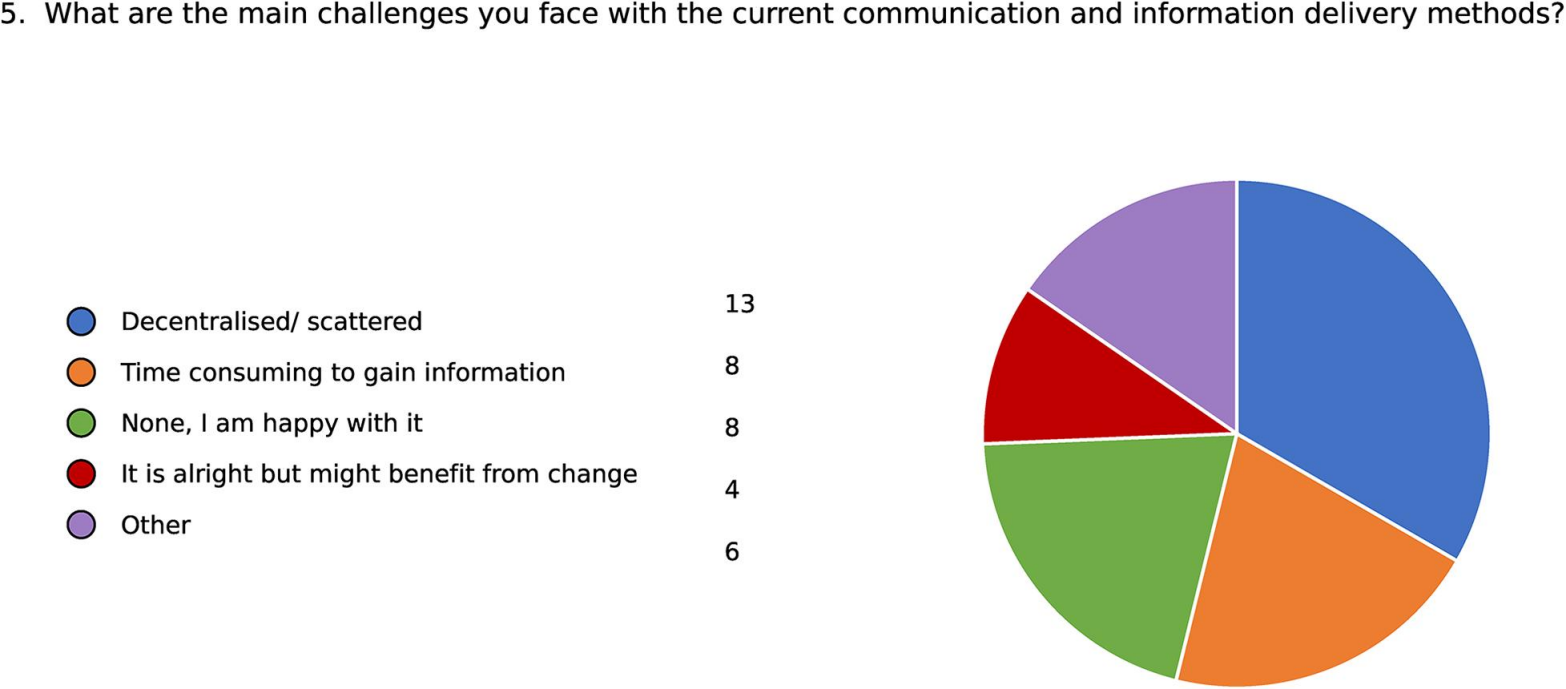
Main challenges reported with current communication and information delivery methods (Source: The researcher).

The systematic evaluation provided validation of the research hypotheses using quantitative and qualitative evidence. H1 (communication method barriers) was confirmed, with survey results demonstrating that structural barriers affected the majority of professionals (decentralisation: 33.3%; time consumption: 20.5%) (Figure 10), with only 12.8% rating current methods as excellent and 74.4% reporting suboptimal experiences. H2 (literature gap) was confirmed through systematic review, with zero MAT-specific chatbots identified across 14 high-quality studies, indicating a significant absence of addiction treatment focus despite available advanced technologies. H3 (enhancing potential) was partially confirmed, with technical feasibility demonstrated through successful prototype development and moderate performance metrics indicating improvement potential.

RQ1 identified information decentralisation and time consumption as the primary structural barriers affecting MAT professionals. RQ2 confirmed the absence of MAT-specific chatbot research, representing a clear gap for future contribution. RQ3 demonstrated that LLM+KG+RAG integration is technically feasible at prototype stage, with performance metrics establishing a baseline for further development.

Overall, the results indicate successful achievement of the research objectives and provide a foundation for continued development. The findings position this work as a contribution to healthcare AI research with practical relevance to MAT delivery in Scotland, subject to the limitations outlined in Section 5.2.

## 4 Discussion

This study addressed the research questions by identifying barriers faced by Medication-Assisted Treatment (MAT) healthcare professionals in accessing MAT standards, revealing gaps in the literature regarding the use of healthcare chatbots for MAT delivery, and demonstrating the feasibility of a prototype chatbot integrating Large Language Models (LLMs), Retrieval-Augmented Generation (RAG), and Knowledge Graphs (KGs) to enhance access to and interpretation of MAT standards. The development of this AI-driven chatbot for MAT in Scotland represents a meaningful step toward addressing limitations in current information dissemination approaches within addiction treatment services.

The choice of a chatbot format was motivated by the need to pragmatically address persistent gaps in access to MAT standards while leveraging emerging technologies in a forward-looking manner. This approach is not intended to substitute existing artefacts such as policy documents, websites, or guidance leaflets, but rather to complement them by addressing a practical information-access problem. MAT standards are not static or standalone policies. They are living documents that evolve in response to emerging evidence, service redesign, and policy updates. In this context, reliance on static formats can limit timely access to current guidance.

**Figure 11:**
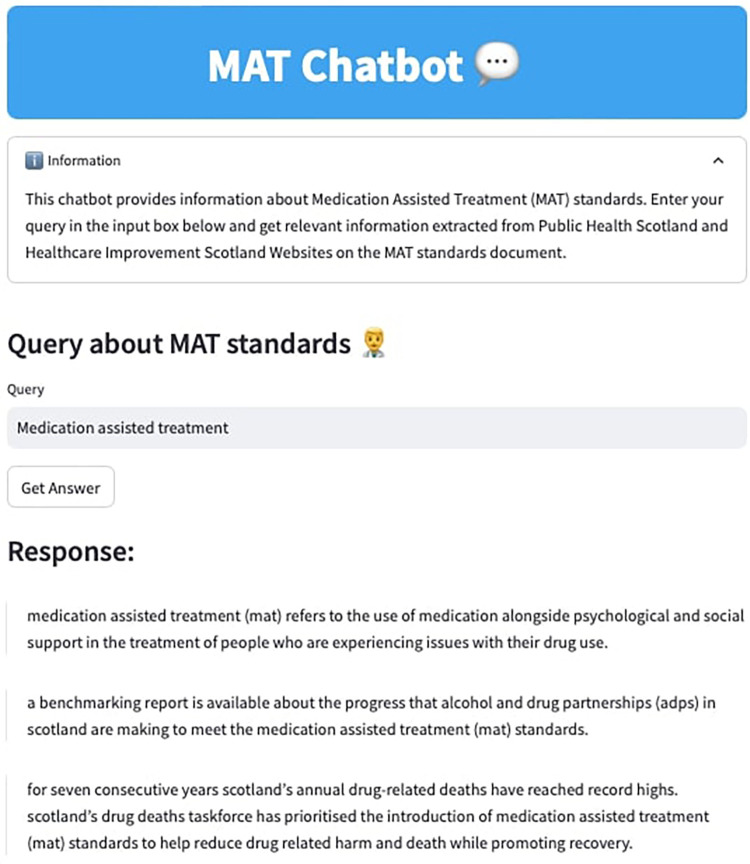
Example interaction MAT chatbot prototype (Source: The researcher).

A conversational chatbot enables clinicians to retrieve context-specific, authoritative information on demand, supporting “at-the-fingertips” access to up-to-date standards (Figure 11). By facilitating natural language queries, the chatbot format reduces search friction and cognitive load, enabling healthcare professionals to navigate evolving MAT guidance more efficiently within real-world clinical and service-planning workflows.

The system is intended to be accessed on demand via a web-based interface, supporting clinicians, commissioners, and service staff during case discussions, training activities, and service design tasks rather than replacing formal documentation or clinical judgement. In this way, the chatbot functions as a decision-support and information-access tool that aligns with existing professional practices and governance structures.

**Figure 12:**
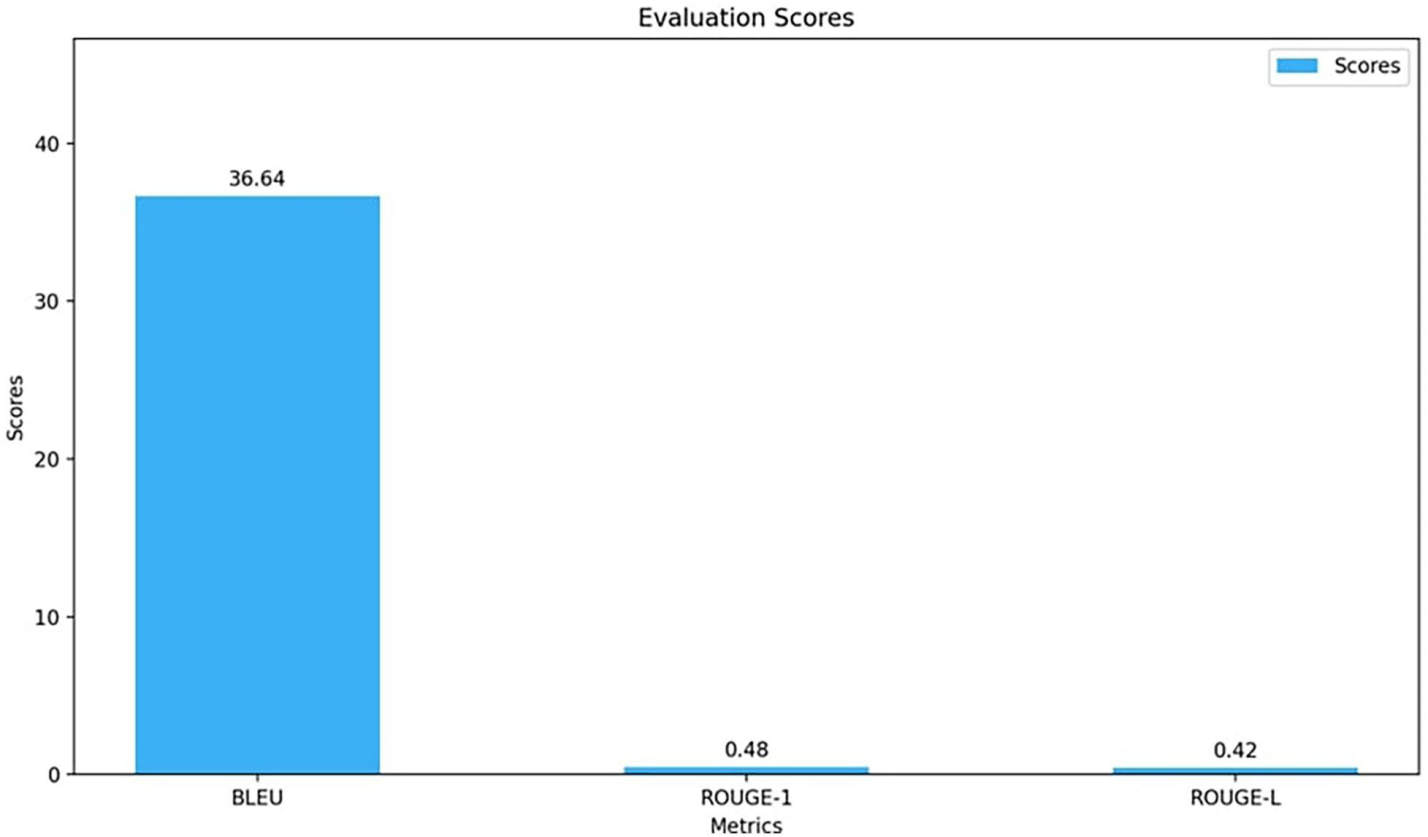
MAT chatbot evaluation (Source: The researcher).

The study’s findings underscore the potential of AI-driven conversational systems to enhance healthcare information access while also highlighting areas that require further development and critical consideration. From a Natural Language Processing (NLP) perspective, the chatbot’s moderate performance, as reflected in BLEU and ROUGE scores (Figure 12), indicates that the prototype successfully captured core linguistic patterns relevant to MAT standards but remains limited in its ability to fully represent semantic nuance. This aligns with broader challenges in NLP, particularly in domain-specific healthcare applications, where precision, safety, and interpretability constrain purely generative performance. Future iterations should therefore focus on refining domain adaptation strategies to better capture the complexity of MAT-related discourse.

From a Machine Learning (ML) standpoint, the chatbot demonstrates promise in its capacity to support timely access to information and personalised responses. However, the reliance on simulated interactions rather than real-world clinical deployment represents a key limitation. This gap between controlled evaluation environments and practical clinical use is a recognised challenge in ML research and reinforces the need for future validation within authentic healthcare settings to assess robustness, generalisability, and real-world impact.

In terms of Knowledge Representation, the structured knowledge graph underpinning the chatbot provides a strong foundation for organising and retrieving MAT standards. The ability of the system to address fragmentation in existing information sources suggests effective schema design and entity–relationship modelling. Nonetheless, expanding the knowledge base remains a priority, particularly through the inclusion of insights derived from individuals with lived and living experience of substance use. Such experiential knowledge can enrich contextual relevance but must be integrated carefully to ensure ethical data governance, privacy protection, and alignment with clinical standards (54).

The Information Retrieval capabilities of the chatbot also warrant further refinement. While initial results indicate effective retrieval of relevant MAT guidance, complex and multi-faceted clinical queries present ongoing challenges. Addressing these challenges will require enhanced retrieval strategies, improved context ranking, and iterative testing with representative user scenarios. The emphasis on more extensive user testing is consistent with best practices in healthcare information systems and highlights the importance of aligning system responses with real-world decision-making needs.

Human Computer Interaction (HCI) considerations are central to the successful adoption of clinical decision-support tools. The chatbot’s conversational interface was designed with usability in mind, and its alignment with established models such as the Technology Acceptance Model suggests potential for acceptance among healthcare professionals, (55). However, usability perceptions in controlled environments do not fully predict sustained use in practice. Broader evaluation involving real-world workflows, varied professional roles, and longitudinal use will be necessary to assess ease of use, trust, and integration into routine practice. The potential extension of chatbot access beyond healthcare professionals to patients and families also introduces additional HCI challenges that require careful design and ethical consideration.

Taken together, these findings reinforce the growing evidence that healthcare chatbots can play a valuable role in supporting information access and professional practice. This study makes a distinct contribution by focusing specifically on Medication-Assisted Treatment and by tailoring the chatbot to the Scottish healthcare context. Unlike general-purpose healthcare chatbots, the proposed system addresses a clearly defined information-access problem arising from fragmented MAT guidance and evolving standards. By streamlining access to authoritative information, the chatbot has the potential to reduce cognitive burden and improve consistency in the interpretation of MAT standards.

The practical implications of this work extend to multiple stakeholder groups. For MAT healthcare professionals, the chatbot offers a tool to rapidly access and interpret standards, supporting informed decision-making and service delivery. For policymakers and service planners, the prototype demonstrates the feasibility of AI-enabled approaches to improving guideline dissemination and may inform future digital investment and implementation strategies. For developers and researchers, the study provides a replicable methodological framework for designing domain-specific healthcare chatbots that integrate LLMs, Knowledge Graphs, and Retrieval-Augmented Generation. Collectively, these contributions position the chatbot as a promising foundation for future research and development aimed at improving access to evolving clinical standards in addiction treatment.

The chatbot is intended to be a knowledge assistant rather than a replacement for clinical judgement, formal documentation, or professional accountability. Its aim is to support access to nationally approved MAT guidance, alongside documented nuances and recommendations that arise through policy consultation and service implementation. When such scenario-based guidance is appropriately documented and incorporated into the knowledge base, the chatbot can make this information accessible in a single, consolidated interface, supporting consistent interpretation across professional roles.

The trustworthiness and clinical value of the system are therefore dependent on the quality, completeness, and currency of the underlying data. There remains a risk that incomplete or outdated information could be relied on inappropriately if content is not regularly reviewed, updated, and fine-tuned. To mitigate this, system design emphasizes transparent sourcing, clear scope definition, and frequent knowledge base updates. Bias and fairness considerations are particularly salient in addiction care, where stigma, vulnerability, and structural inequalities persist. Although Scottish MAT standards are co-produced through consultation with clinicians, people with lived and living experience, and third-sector organisations, future development should continue to prioritise inclusive co-production and bias-aware design to ensure AI-supported tools do not inadvertently reinforce inequity or exclusion.

Conversational AI introduces misinterpretation risks that structured guideline documents do not. The primary risks include paraphrased responses that subtly alter clinical meaning, responses that conflate related but distinct standards, and outputs that appear confident for queries outside the knowledge base scope. The RAG architecture mitigates these risks by grounding responses in retrieved source documents rather than relying solely on LLM generation, and the knowledge graph provides structured relationship context that constrains response generation. The system includes explicit disclaimers stating it is for informational and decision-support purposes only and should not substitute professional clinical judgement. Nonetheless, residual risks remain and human oversight will be essential in any future deployment context.

## 5 Conclusions

### 5.1 Development challenges and technical constraints

The collection of MAT data presented significant challenges due to the fragmented nature of information distribution across multiple websites and ethical constraints that prevented scraping from certain authoritative sources. These limitations substantially reduced the scope and depth of training data available for the chatbot’s knowledge base, potentially introducing biases and knowledge gaps that could affect system performance and clinical utility. The integration of diverse datasets into a cohesive knowledge graph proved technically complex, requiring sophisticated data harmonization approaches to maintain semantic consistency across heterogeneous information sources.

Limited computational resources significantly constrained the full integration potential of advanced technologies including Llama2, Neo4j, and Retrieval-Augmented Generation (RAG). These sophisticated AI technologies require substantial computational power and specialized technical expertise, while the restricted dataset further limited their optimization potential within the prototype development framework (49). The exploration of document retrieval approaches as more computationally feasible alternatives demonstrated the necessity of balancing technological ambition with practical implementation constraints, particularly relevant given the computational demands of state-of-the-art language models.

Fine-tuning the language model was explored but not fully realised due to computational resource constraints. Domain adaptation was therefore achieved through the RAG pipeline, which retrieves MAT-specific context at inference time. These NLP challenges reflect broader issues in developing specialized healthcare AI applications, where domain-specific performance requirements must be balanced against computational efficiency and deployment feasibility within healthcare IT infrastructure constraints. Creating a functional yet intuitive user interface while maintaining optimal system response times presented additional design challenges, particularly when integrating multiple advanced AI technologies with the need to support healthcare professional workflow requirements.

Ethical considerations and data privacy regulations substantially influenced data collection methodologies and chatbot development approaches, consistent with growing concerns about responsible AI deployment in healthcare settings (56). Future integration into healthcare systems will necessitate strict adherence to ethical guidelines, robust data protection measures, and comprehensive compliance with regulations including GDPR and healthcare-specific governance frameworks. The implementation of thorough Data Protection Impact Assessment (DPIA) protocols will be essential for healthcare system deployment, requiring transparent consent mechanisms and comprehensive user trust maintenance strategies.

### 5.2 Research limitations

As participation was voluntary and the response rate was approximately 31%, it is possible that respondents were more engaged with MAT communication challenges than non-respondents. This may limit generalisability, and findings should be interpreted as indicative of user needs rather than representative of all MAT professionals.

A key limitation of this study is that not all Scottish MAT Standards could be incorporated into the knowledge base due to access restrictions imposed by website robots.txt policies. As a result, the chatbot’s responses are limited to publicly accessible guidance and may not reflect the full breadth of current MAT documentation, reinforcing the prototype nature of the system and informing priorities for future development.

The ethical data collection approach, while maintaining research integrity and regulatory compliance, inherently limited the dataset’s breadth and depth, potentially affecting the chatbot’s comprehensiveness and clinical accuracy. The exclusion of certain authoritative data sources due to access restrictions and the challenges encountered in integrating diverse dataset formats further contributed to knowledge base limitations. These constraints reflect the tension between responsible research practices and optimal system performance, highlighting the need for collaborative approaches with healthcare organizations to enable more comprehensive data access while maintaining ethical standards.

The moderate BLEU and ROUGE scores (36.64 and 0.48/0.42 respectively) obtained using established evaluation metrics (50, 51) indicate challenges in capturing semantic nuances and contextual subtleties, particularly for complex clinical queries requiring sophisticated domain knowledge. These automated metrics, while providing standardized evaluation benchmarks used in healthcare chatbot research (36), may not fully capture the clinical utility and professional appropriateness essential for healthcare applications. The limitation of evaluation primarily to automated metrics rather than comprehensive clinical assessment represents a significant constraint on generalizability and practical applicability determination. Additionally, the entity recognition accuracy reported reflects expert validation against source documentation rather than formal benchmarking against a pre-existing annotated corpus, which represents a further constraint on the generalisability of the evaluation findings.

Limited user feedback collection and the absence of real-world deployment testing restrict the generalizability of findings and limit understanding of practical implementation challenges. The prototype evaluation within controlled research environments may not adequately reflect the complexities and demands of actual clinical workflows and healthcare system integration requirements. Extensive testing within authentic healthcare settings will be essential to identify and address usability issues, workflow integration challenges, and professional acceptance barriers that cannot be anticipated through laboratory-based evaluation approaches, particularly given the documented challenges in MAT information access reported by healthcare professionals (3).

Limited computational resources and development timeline constraints significantly impacted system scalability potential and real-time performance optimization. These limitations also restricted opportunities for broader user feedback collection and iterative refinement based on diverse healthcare professional perspectives and workflow requirements. The resource constraints encountered highlight the importance of realistic project scoping for healthcare AI research while identifying specific infrastructure requirements for successful scaling and implementation within standard healthcare IT environments.

### 5.3 Future research directions

Building on the limitations identified, several directions for future research and system development emerge, particularly in relation to how nationally governed MAT standards are represented, contextualised, and extended within AI-supported tools. It is important to note that the Medication-Assisted Treatment standards published by Public Health Scotland are developed through structured national governance processes involving the MAT Implementation Support Team and relevant policy stakeholders. These processes include consultation with clinicians, individuals with lived and living experience of substance use, families and carers, and third-sector organisations, with proposed adjustments reviewed, approved, and formally signed off prior to publication. As such, national MAT standards represent a co-produced synthesis of diverse perspectives rather than a purely top-down policy view.

However, while this consultative approach strengthens the legitimacy and consistency of national guidance, the formal publication process may still abstract away local service-level nuances, informal practices, and evolving frontline experiences. The ideal future scenario is therefore one in which nationally approved MAT standards are complemented by mechanisms that surface contextual insights from local services and communities in a structured and governance-aligned manner. Integrating these perspectives alongside authoritative national guidance represents an important direction for future development.

Future development should prioritize systematic engagement with MAT healthcare professionals and individuals with lived and living experiences of substance use disorders to enrich the dataset and ensure chatbot responses remain contextually relevant and grounded in authentic clinical scenarios (54). This collaborative approach will be essential for developing culturally appropriate and clinically meaningful AI applications that address the comprehensive needs of individuals with heroin addiction as outlined in Scotland’s ten MAT standards (9). The integration of diverse stakeholder perspectives, including patients, families, and community organizations, will enhance the system’s relevance and acceptance while ensuring that technological solutions address genuine rather than perceived healthcare needs.

While this study demonstrates technical feasibility and promising performance, usability testing was limited to prototype-level evaluation rather than real-world clinical deployment. Building on the need for stakeholder engagement, future work should expand usability evaluation within authentic clinical environments, incorporating structured feedback from MAT healthcare professionals to assess workflow integration, interpretability, and practical utility. Where live deployment is not immediately feasible, simulation-based case scenarios using representative MAT pathways could provide a controlled and ethical approach to evaluating task success, decision support effectiveness, and clinical relevance.

Beyond technical performance, real-world deployment of AI-driven clinical decision support tools faces practical barriers that warrant further investigation. Interoperability with existing healthcare IT systems remains a key challenge, particularly given the heterogeneity of data standards and infrastructures across NHS settings. Integration with Electronic Health Records (EHRs) would be essential for seamless clinical workflow adoption but introduces governance, security, and interoperability constraints that extend beyond prototype development. In addition, effective staff training and change management would be critical to ensure appropriate use, mitigate over-reliance on automated outputs, and build clinician trust in AI-assisted decision support tools. Addressing these organisational and socio-technical barriers will be central to successful real-world implementation.

Comprehensive expansion of the dataset to include broader MAT information sources, regular knowledge base updates, and integration of diverse data formats including PDF documents will be necessary for clinical deployment readiness. The development of automated update mechanisms will ensure that the chatbot maintains currency with evolving treatment guidelines and evidence-based practices, addressing the dynamic nature of addiction treatment protocols. Advanced data integration approaches should be explored to incorporate real-time information feeds from authoritative healthcare sources while maintaining data quality and clinical accuracy standards essential for supporting clinical decision-making in Scotland’s ongoing drug-related death crisis (7). A production-scale deployment would additionally require migration to a more robust database infrastructure beyond the prototype implementation, with automated content ingestion pipelines and periodic benchmark testing at larger graph sizes identified as priorities for future development.

Continued exploration of sophisticated AI techniques, including larger parameter language models, enhanced knowledge graph architectures, and optimized Retrieval-Augmented Generation implementations, will be essential for improving accuracy, reliability, and conversational naturalness. The successful applications of similar technologies in other healthcare domains, such as ophthalmology (28) and rheumatological care (27), suggest substantial potential for advancement in addiction treatment applications. The development of hybrid approaches that combine multiple AI technologies while maintaining computational efficiency will be crucial for practical healthcare system implementation within NHS Scotland infrastructure constraints. Implementation of confidence thresholding and scope-boundary detection to handle queries outside the knowledge base would further reduce hallucination risk and is identified as a development priority.

Future research should implement broader evaluation methodologies incorporating diverse performance metrics, extensive user testing protocols, and longitudinal assessment within authentic healthcare settings. This should include semantic evaluation metrics such as BERTScore alongside structured human clinical assessment to provide richer evaluation of response quality and clinical appropriateness beyond the lexical overlap measures used in this prototype evaluation. An ablation study comparing retrieval precision with and without RAG integration would provide quantitative evidence of the RAG component’s specific contribution and is identified as a methodological priority. The development of healthcare-specific evaluation frameworks that prioritize clinical utility alongside technical performance will be essential for meaningful system assessment. Comparative effectiveness studies measuring information retrieval time and accuracy against existing workflows will provide quantitative evidence of practical benefit and important support for healthcare system adoption decisions and resource allocation justification, particularly important given the identified barriers in current MAT information delivery methods.

The implementation of comprehensive frameworks for continuous learning and system adaptability will be crucial for maintaining long-term effectiveness and clinical relevance. These mechanisms should incorporate regular content updates, user feedback integration, and performance monitoring to ensure sustained value within evolving healthcare environments. Automated learning approaches that enable system improvement through user interaction analysis while maintaining privacy and security standards represent important development priorities for future iterations, building upon the foundation established through this prototype development.

Ongoing attention to ethical implications and potential algorithmic biases will be essential throughout future development phases, particularly given the stigmatization challenges facing individuals with substance use disorders. The prioritization of user trust, informed consent, and responsible AI deployment practices must remain central to all enhancement efforts. Comprehensive bias detection and mitigation strategies should be implemented to ensure equitable access and appropriate responses across diverse healthcare populations and clinical scenarios.

### 5.4 Research contributions

This study demonstrated the feasibility of developing a prototype chatbot capable of enhancing access to Medication-Assisted Treatment (MAT) standards among healthcare professionals in Scotland, addressing gaps identified in both literature and practice. The findings validate the potential for AI-driven solutions to address the information access barriers documented among Scottish healthcare professionals, where professionals identified decentralisation (33.3%) and time-consuming retrieval processes (20.5%) as major barriers. Through the integration of Large Language Models (LLMs), Retrieval-Augmented Generation (RAG), and Knowledge Graphs (KGs), this research provides proof-of-concept evidence for technological approaches that could improve the dissemination and utilisation of MAT information in clinical practice. The implementation of Neo4j knowledge graph technology with 227 entities and 136 relationships achieved structured representation of Scotland’s ten MAT standards while maintaining query performance suitable for prototype-stage evaluation.

The mixed-methods approach, incorporating quantitative surveys of 39 healthcare professionals and a systematic literature review following PRISMA guidelines (35), aligns with pragmatic research paradigms that prioritise practical solutions to identified healthcare challenges. The systematic literature review identified an absence of MAT-specific applications among 14 high-quality healthcare chatbot studies, representing a gap in research attention to addiction treatment despite the proven effectiveness of MAT interventions in reducing drug-related mortality (9). Despite moderate performance metrics reflecting the prototype’s early development stage, the demonstrated feasibility and identified enhancement pathways indicate potential for improving MAT standards delivery. The system’s BLEU score of 36.64 and ROUGE scores of 0.48 and 0.42 establish a quantitative baseline for further development while indicating clear directions for improvement.

This research developed what is, to our knowledge, the first chatbot tailored specifically to Scottish MAT healthcare professionals, addressing the fragmented information landscape that currently requires professionals to navigate multiple unintegrated sources. The system was designed to address the multi-source dependency identified among survey respondents, who reported accessing an average of 2.5 different sources for comprehensive MAT information.

The demonstration of multi-technology AI integration within healthcare-specific constraints provides methodological insights applicable to broader healthcare AI development. The evidence-based development approach, grounded in systematic needs assessment, establishes a replicable framework for creating clinically relevant AI applications that address identified healthcare challenges. The transition from research prototype to a fully implemented healthcare system requires substantial additional investigation and refinement. The identified limitations, including restricted dataset scope and technological constraints, underscore the necessity for continued research to ensure long-term effectiveness and clinical adaptability within Scotland’s healthcare infrastructure and regulatory framework.

Future research priorities should address the integration of advanced AI technologies to enhance functional capabilities while addressing ethical, social, and organisational implementation challenges. This includes establishing data governance frameworks, developing integration strategies within existing healthcare systems, and creating user training infrastructure that respects the workflow requirements of MAT healthcare professionals. Subsequent iterations should incorporate real-time content updates, electronic health record integration for personalised clinical recommendations, and expanded coverage of the broader substance use disorder spectrum. Scotland’s drug-related death rate of 267 per million people, second highest globally (1), underscores the public health urgency of continued development in this area.

This research advances understanding of AI applications in healthcare by demonstrating that LLM, RAG, and knowledge graph technologies can be integrated into a functional prototype addressing a confirmed clinical information gap. The findings provide a documented foundation for future development toward more sophisticated, clinically validated solutions for MAT standards delivery in Scotland.

## Data Availability

The anonymised survey data supporting the conclusions of this article will be made available by the authors on reasonable request. The text corpus used for the knowledge base was compiled from publicly available web pages of Public Health Scotland and Healthcare Improvement Scotland. The source code for the chatbot prototype, including the data collection and knowledge graph construction pipeline, is openly available at https://github.com/Snwobi/MATCHATBOT_Repo.
